# Analyzing Corin–BNP–NEP Protein Pathway Revealing Differential Mechanisms in AF-Related Ischemic Stroke and No AF-Related Ischemic Stroke

**DOI:** 10.3389/fnagi.2022.863489

**Published:** 2022-05-09

**Authors:** Xiaozhu Shen, Nan Dong, Yiwen Xu, Lin Han, Rui Yang, Juan Liao, Xianxian Zhang, Tao Xie, Yugang Wang, Chen Chen, Mengqian Liu, Yi Jiang, Liqiang Yu, Qi Fang

**Affiliations:** ^1^Department of Neurology, The First Affiliated Hospital of Soochow University, Suzhou, China; ^2^Department of Geriatrics, Lianyungang Second People’s Hospital, Lianyungang, China; ^3^Department of Neurology, Suzhou Industrial Park Xinghai Hospital, Suzhou, China; ^4^Department of General Medicine, Lianyungang Hospital, Affiliated to Jiangsu University (Lianyungang Second People’s Hospital), Lianyungang, China; ^5^Bengbu Medical College, Bengbu, China

**Keywords:** atrial fibrillation, cardioembolism, B-type natriuretic peptide, Corin peptide, neprilysin, DNA methylation

## Abstract

**Background:**

The incidence of atrial fibrillation (AF)-related stroke increases with aging. Natriuretic peptides (NPs) family, including Corin-B type natriuretic peptide (BNP)-neprilysin (NEP) protein levels increased with age and are risk markers of cardiovascular and cerebrovascular diseases, such as AF and cardioembolic stroke. Aging is also linked to epigenetics, specifically DNA methylation. However, only a few studies have investigated the effect of DNA methylation on the NP system. Thus, the present study aimed to investigate whether the Corin-BNP-NEP protein pathway is involved in the pathogenesis of AF-stroke and CpG methylation in the promoter region of the Corin protein gene has an effect on AF-related ischemic stroke.

**Methods:**

A total of 82 patients hospitalized with acute ischemic strokes were enrolled in this study. The differences in clinical information were compared between the AF-stroke (*n* = 37) and no AF-stroke groups (*n* = 45). Plasma-soluble Corin and NEP were detected using an ELISA kit. CpG methylation in the promoter region of the gene was assessed by a next-generation sequencing-based bisulfite sequencing polymerase chain reaction (BSP).

**Results:**

(1) Patients in AF-stroke were older, had higher initial NIHSS score, 90-day mRs, higher D2-dimer, INR, and APTT, and low TG, TC, and HbA1c (all *p* < 0.05). (2) Serum levels of Corin and BNP in the AF-stroke group were significantly higher than that in the no AF-stroke group (*p* < 0.05). No significant difference was detected in the serum levels of NEP between the two groups. (3) The levels of CpG methylation in the promoter region of the Corin protein gene in the AF-stroke group was significantly lower than that in the no AF-stroke group (*p* < 0.05). The CpG sites with maximal methylation differences between the two groups were CORIN:678, CORIN:682, CORIN:694, and CORIN:700.

**Conclusion:**

The current findings raise the possibility that the Corin–BNP–NEP protein pathway may be involved in the pathogenesis of AF-related ischemic stroke. Deficient CpG methylation in the promoter region of the Corin protein gene is associated with AF-related ischemic stroke.

## Introduction

In recent years, the aging of the global population has led to an increased burden of disease and disability. A large-scale population-based national stroke survey shows that the burden of stroke in China has been increasing over the past 30 years, and ischemic stroke constituted 69.677.8% incidence and prevalence of strokes (Wang et al., 2017). Hitherto, several classification methods are available for ischemic stroke. Using the Trial of Org 10172 in acute stroke treatment (TOAST) criteria, ischemic stroke can be divided into large artery atherosclerosis (LAA), cardioembolism (CE), small artery occlusion (SAO), stroke of other determined etiology (OC), and stroke of undetermined etiology (SUD) (Liu et al., 2020). CE stroke accounts for 14–30% of all cerebral infarctions (Maida et al., 2020). AF is one of the main causes of CE stroke. It is age-related, and AF-associated ischemic strokes have a high rate of disability and mortality (Zhou et al., 2016; GBD 2016 Causes of Death Collaborators, 2017). The prevention and treatment of AF-associated ischemic stroke are crucial for healthy aging.

For a prolonged period, the circulating levels of natriuretic peptides (NPs) have been widely used as clinical biomarkers of cardiovascular function. B-type natriuretic peptide (BNP) is a hormone belonging to the natriuretic peptide family that retains a common ring structure and conserved amino acid sequence. BNP is cleaved from the proBNP precursor by enzymatic processing between amino acid residues 76 and 77, similar to the amino-terminal portion of proBNP (i.e., NT-proBNP), and this enzymatic reaction was undertaken by the enzyme Corin (Yandle and Richards, 2015). Several enzymes are involved in NP degradation, among which Neprilysin (NEP) plays a dominant role. It cleaves BNP at two main positions, of which the cleavage between Met-4 and Val-5 is the primary site within the ring structure between Arg-17 and Ile-18 (Yandle and Richards, 2015; Chen and Burnett, 2017). Corin-BNP-NEP constitutes a protein pathway from generation to decomposition. The plasma levels of BNP (Everett et al., 2015; Yoshida et al., 2019) and the risk markers of cardiovascular and cerebrovascular diseases, such as AF and CE stroke, are increased with age (Berntsson et al., 2014; Goetze et al., 2020). Whether the Corin-BNP-NEP protein pathway is involved in the occurrence of AF-associated stroke is yet to be elaborated.

Epigenetics, specifically DNA methylation (DNAm), is linked to aging (Day et al., 2013; Horvath, 2013; Tharakan et al., 2020). DNAm is the most abundant epigenetic marker in the human genome for controlling gene expression (Belsky et al., 2022). It usually occurs in CpG islands and is mostly in the proximal promoter region of the human genome, which modifies an individual’s biological function by regulating gene expression or genome sequence stability (Deng et al., 2019). DNAm modifications are heterogeneous in terms of organ and tissue components. Abnormal DNAm has been associated with many cardiovascular, cerebrovascular, and other diseases, including Alzheimer’s disease (AD), Parkinson’s disease (PD), ischemic stroke, coronary artery disease (CAD), myocardial infarction, and cancer (Deng et al., 2019; Miao et al., 2019; Martínez-Iglesias et al., 2020; Sharma et al., 2020; Wang et al., 2021).

Previous studies have shown that epigenetics is closely related to aging, which in turn is related to NP. Strikingly, only a few studies have evaluated the effect of DNAm in the NP system. Thus, the present study aimed to investigate whether the Corin–BNP–NEP protein pathway is involved in the pathogenesis of AF-stroke and CpG methylation in the promoter region of the Corin protein gene has an effect on AF-related ischemic stroke.

## Materials and Methods

### Patients and Samples

Subjects were enrolled after obtaining written informed consent and approval from the Ethics Committee of Soochow University. This study recruited patients with first-ever ischemic or hemorrhagic stroke onset within 48 h confirmed by brain computed tomography (CT) or magnetic resonance imaging (MRI) from three hospitals between September 2019 and December 2020. This study was approved by Soochow University (No. 2019-057). The inclusion criteria were as follows: (1) Age ≥ 18 years; (2) Within 24 h of onset, CTA + CTP suggested the presence of infarct core or within 1 week from onset to the time of cranial MRI examination, and acute cerebral infarction lesions were visible on MR DWI sequences; (3) Patients with the first onset of previous cerebral infarction with no significant sequelae left and re-acute onset; (4) Patients who completed ECG or Holter and ECG monitoring during their stay in the hospital. Patients who fulfilled one of the following conditions were excluded: (1) Patients with cerebral hemorrhage and occupancy (emergency head CT excludes cerebral hemorrhage, while post-infarction hemorrhage is not excluded if the patient was hospitalized); (2) Patients with transient ischemic attack; (3) Severe infection or septic shock; (4) History of severe trauma with surgical treatment; (5) significant hepatic and renal insufficiency; (6) Endocrine, immune, and oncological diseases; (7) Pregnancy; (8) other causes of cardiogenic stroke, including patent foramen ovale, left atrial mucinous tumor, rheumatic heart disease, dilated cardiomyopathy, and hypertrophic cardiomyopathy; (9) Other causes that can cause acute multiple foci of infarction, such as vasculitis, coagulation system diseases, and tumor embolism.

Finally, 82 patients were enrolled in this study. Two groups were divided based on whether AF was detected during the course of the disease: the AF-stroke group (*n* = 37) and the no AF-stroke group (*n* = 45). In the no AF-stroke group, large artery atherosclerosis accounted for 100% of the cases, according to TOAST typing.

About 5 ml venous blood was collected from subjects within 4.5 h of onset and before revascularization treatment and stored in EDTA anticoagulation tubes. Of this, 3 ml was frozen at –80°C until sequencing, and the remaining 2 ml was subjected to centrifugation at 5°C, and about 600 ml plasma was obtained and stored at –80°C.

### Basic Data Collection

During enrollment, the medical history was taken, and the routine physical examination of the participants was performed by experienced physicians. Medical history included age, sex, systolic blood pressure (SBP), initial NIHSS score, treatment options (i.e., thrombolysis, embolectomy, bridging therapy, and conservative treatment), and 90-day mRs. Laboratory tests included D2-dimer, INR, PLT, Fib, APTT, Cr, TC, TG, LDL-C, Hcy, Glucose, HbA1c, and TnI.

### Plasma Soluble Corin, B Type Natriuretic Peptide, and Neprilysin Level Tests

Plasma-soluble Corin level was measured using a human CRN ELISA kit (Catalog: IC-CRN-Hu, IC ImmunoClone Inc., Shanghai, China); plasma-soluble NEP was measured using a human NEP ELISA kit (Catalog: JL15469, Jianglai Inc., Shanghai, China); plasma soluble BNP level was collected from clinical data.

### Next-Generation Sequencing-Based Bisulfite Sequencing Polymerase Chain Reaction

Gene-specific DNAm was assessed by BSP, according to a previously published method (Pan et al., 2018). Briefly, BSP primers were designed using the online MethPrimer software, and the sequences of primers were as follows: *CORIN*: Forward 5′-GAAGGAAATTTTGTTTATGATTTTGGGAGGGT-3′ and Reverse 5′-ATAACCTCTTAATCCCRATAAATTCAAAATCAA CC-3′; *CORIN*: Forward 5′-GATTTTTAGGTATTAATTGGG GGTYGGGGAATT-3′ and Reverse 5′- CCTCCAAACATC TAATAAACTTAACTACACAC-3′. An equivalent of 1 μg of genomic DNA was converted using the ZYMO EZ DNA Methylation-Gold Kit (Zymo Research, Irvine, CA, United States) and 0.05% of the elution products were used as templates for PCR amplification with 35 cycles using KAPA 2G Robust HotStart PCR Kit (Kapa Biosystems, Wilmington, MA, United States). For each sample, the BSP products of multiple genes were pooled equally, and 5′-phosphorylated and 3′-dA-tailed products were ligated to the barcoded adapter using T4 DNA ligase (NEB). The barcoded libraries from all samples were sequenced on the Illumina platform.

For the bisulfite sequencing reads of each sample, firstly, adapters and low-quality reads were removed using Trimmomatic-0.36 software. After removing the adapter sequences and filtering out the low-quality reads, the clean sequencing reads were aligned to the target sequences using Bsmap (v2.73) software with the default parameters, which combines genome hashing and bitwise masking to achieve fast and accurate bisulfite mapping. Methylation levels were defined as the fraction of read counts of “C” in the total read counts of both “C” and “T” for each covered C site. Based on such read fraction, methylated cytosine was called using a binomial distribution to compute the probabilistic mass function for each methylation context (CpG). Two-tailed Fisher’s exact test was used to identify the cytosines that are differentially methylated between two samples or groups. Only those CpGs covered by a minimum of 200 reads in at least one sample were considered for testing.

### Statistical Analysis

Data were analyzed using the SPSS software (IBM SPSS Statistics for Windows, version 25.0; IBM Corp., Armonk, NY, United States), GraphPad Software (GraphPad Prism for Windows, version 9.0.0; San Diego, CA, United States), and R software package version 4.1.2.^1^ *p* < 0.05 in a two-tailed test indicated statistical significance. Baseline information included in the analysis included age, sex, SBP, initial NIHSS score, treatment options (i.e., thrombolysis, embolectomy, bridging therapy, and conservative treatment), 90-day mRs, D2-dimer, INR, PLT, Fib, APTT, Cr, TC, TG, LDL-C, Hcy, glucose, HbA1c, and TnI. The Kolmogorov–Smirnov test was used to assess the normality of numerical variables. Mann–Whitney *U*-tests were used for analysis in the case of non-normal distribution, described by median and quartile range (IQR). The continuous variables of normal distribution were analyzed by independent sample *t*-test and expressed as mean ± standard deviation (SD), and chi-squared test or Fisher exact test for categorical variables. Mann–Whitney *U*-test was used to assess the difference in the plasma levels of Corin, BNP, and NEP between the two groups. Based on the absolute coordinates of the detected gene region, the map shows the average methylation level of each site in each sample and is labeled with different colors according to the biological groups, using the formula model function in R language to simulate the combined trend line. Cluster analysis was performed to assess the methylation levels of CpG sites in all samples and display the categorical correlation of methylation levels between samples and sites in the form of heat maps. The color changes from blue to red indicated a gradually increasing methylation level. Then, the average methylation level of each CpG site in each sample was evaluated using box plot + bee colony plot to show the methylation distribution of each region between the AF-stroke and no AF-stroke groups and analyze the difference between the two groups by the Wilcox test.

## Results

### Clinical Characteristics of the Participants

The clinical parameters of all participants are summarized in Table 1. Compared to the no AF-stroke group, patients in the AF-stroke were older, had higher initial NIHSS score and 90-day mRs, higher D2-dimer, INR, APTT, and lower TG, TC, and HbA1c (all *p* < 0.05). In addition, no statistical difference was observed in gender, SBP, PLT, Fib, Cr, LDL-C, Hcy, glucose, and TnI between the two groups (all *p* > 0.05).

**TABLE 1:** Clinical characteristics of the participants.

Characteristics	AF-stroke (*n* = 37)	noAF-stroke (*n* = 45)	T/U/X^2^	*Z*	*p*-value
Median Age (IQR)—year	73(64.5, 82.5)	65(56.5, 74)	573	-2.42	0.016
Sex—no. (%)			2.821		0.093
Male	20(54.1)	16(35.6)			
Female	17(45.9)	29(64.4)			
Median SBP(IQR)—mmHg	144(130.5, 162.5)	154(137.5, 172.5)	657.5	-1.631	0.103
Initial NIHSS score—no. (%)			19.326		<0.001
0–5	4(10.8)	24(53.3)			
6–14	18(48.6)	15(10.8)			
≥15	15(40.5)	6(13.3)			
Treatment options—no. (%)			14.375		0.002
Thrombolysis	17(45.9)	36(80)			
Embolectomy	8(21.6)	1(2.2)			
Bridging therapy	4(10.8)	1(2.2)			
conservative treatment	8(21.6)	7(15.6)			
90-day-mRS—no. (%)			15.62		0.016
0	5(13.5)	16(35.6)			
1	4(10.8)	6(13.3)			
2	1(2.7)	5(11.1)			
3	4(10.8)	5(11.1)			
4	12(32.4)	11(24.4)			
5	6(16.2)	1(2.2)			
6	5(13.5)	1(2.2)			
Median D2-dimer (IQR)—mg/L	1.06(0.60, 1.93)	0.39(0.25, 0.85)	443.5	-3.629	<0.001
Median INR(IQR)	1.10(1.05, 1.16)	1.04(1.02, 1.07)	432.5	-3.735	<0.001
Mean PLT(SD)—^10^9^/L	190.89 ± 98.23	218.49 ± 45.91	1.573		0.122
Median Fib (IQR)—g/L	3.20(2.60, 4.15)	3.28(2.59, 3.96)	798	-0.322	0.748
Median APTT(IQR)—S	34.4(31.5, 37.9)	32.3(28.05, 36.3)	610.5	-2.069	0.039
Median Cr (IQR)—mmol/L	66.20(54.10, 85.95)	66.20(53.20, 77.40)	745	-0.815	0.415
Median TC(IQR)—mmol/L	3.84(3.20, 4.40)	4.31(3.58, 5.27)	611	-2.064	0.039
Median TG(IQR)—mmol/L	0.99(0.73, 1.31)	1.17(0.93, 1.60)	617.5	-2.004	0.045
Mean LDL-C(IQR)—mmol/L	2.37(1.73, 2.91)	2.61(1.93, 3.27)	699	-1.524	0.128
Median Hcy(IQR)—mmol/L	12.10(9.25, 17.00)	9.40(8.75, 12.50)	637	-1.823	0.068
Median Glucose (IQR)—mmol/L	6.38(5.50, 7.67)	7.00(5.50, 9.55)	697	-1.263	0.207
Median HbA1c(IQR)—%	5.60(5.30, 6.35)	6.30(5.75, 7.95)	521	-2.905	0.004
Median TnI(IQR)—μg/L	14.70(9.75, 30.37)	11.26(7.29, 18.67)	628.5	-1.901	0.057
Median Corin (IQR)—ng/ml	8.17(4.68, 30.62)	4.93(2.12, 9.01)	612	-2.055	0.040
Median BNP(IQR)—pg/ml	269.85(64.54, 633.90)	13.27(0, 72.12)	342.5	-4.624	<0.001
Median NEP(IQR)—pg/ml	130.5(8.63, 274.88)	131.1(3.57, 253.94)	789	-0.407	0.684

*SBP, systolic pressure; DBP, diastolic pressure; NIHSS, National Institutes of Health Stroke Scale; BMI, body mass index; 90-day-mRs, 90-day -Modified Rank in Scale.*

### Schematic of the Methylation Level Distribution of Each CpG Site

**FIGURE 1:**
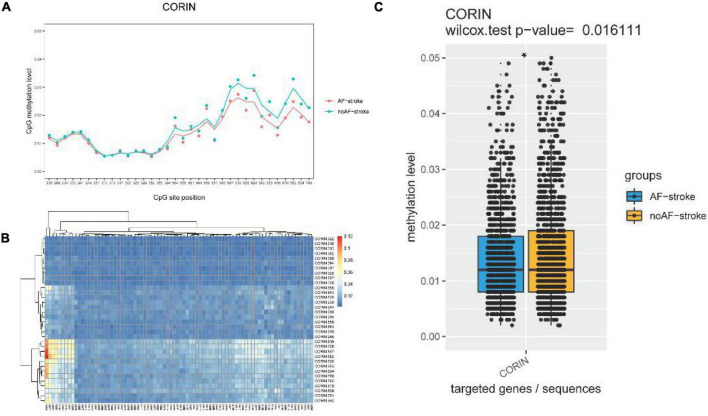
**(A)** Schematic diagram of the methylation level distribution of each CpG site of group AF-stroke and group noAF-stroke in the target area. Based on the absolute coordinates of the detected gene region, the map shows the average methylation level of each site in each sample and is labeled with different colors according to biological groupings, using the formula model function in R language to simulate the combined trend line. The sites with the greatest methylation differences between the two groups are CORIN:678, CORIN:682, CORIN:694, and CORIN:700. **(B)** Methylation level clustering heat map of all samples in group AF-stroke and group noAF-stroke. Perform cluster analysis on the methylation levels of CpG sites in all samples, and display the categorical relationship of methylation levels between samples and sites in the form of heat maps. The color changes from blue to red, indicating that the methylation level is gradually increasing. **(C)** Comparison of CpG methylation levels in target areas between group AF-stroke and group noAF-stroke. Count the average methylation level of each CpG site in each sample, use box plot + bee colony plot to show the methylation distribution of each region between group AF-stroke and group noAF-stroke, and analyze the difference between these two groups by the Wilcox test. And there was a significant difference between these two groups, *p < 0.05.

The schematic of the methylation level distribution of each CpG site of the AF-stroke and no AF-stroke groups in the promoter region of the Corin protein gene as shown in Figure 1A. Based on the absolute coordinates of the detected gene region, the map shows the average methylation level of each site in each sample and is labeled with different colors according to biological groupings, using the formula model function in R language to simulate the combined trend line. The CpG sites with the maximal methylation differences between the two groups are CORIN:678, CORIN:682, CORIN:694, and CORIN:700, respectively.

### Methylation Level Clustering Heatmap of All Samples

The methylation level clustering heatmap of all samples in the AF-stroke and no AF-stroke groups is shown in Figure 1B. Cluster analysis was performed on the methylation levels of CpG sites in all samples, which displayed the categorical correlation of methylation levels between samples and sites in the form of heatmaps. The color changes from blue to red indicated that the methylation level increases gradually.

### Plasma Levels of Corin, B Type Natriuretic Peptide, Neprilysin

**FIGURE 2:**
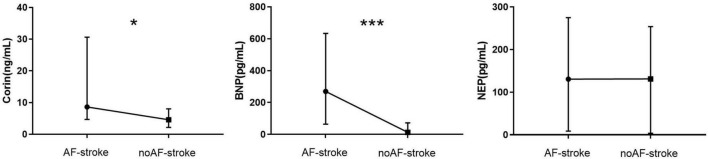
Plasma levels of Corin–BNP–NEP in groups AF-stroke and noAF-stroke. Plasma levels of Corin in AF-stroke group [8.17 (4.68, 30.62) ng/ml] were significantly higher than in noAF-stroke group [4.93 (2.12, 9.01) ng/ml], *p < 0.05. Plasma levels of BNP in AF-stroke group [269.85 (64.54, 633.90) pg/ml] were significantly higher than in noAF-stroke group [13.27 (0, 72.12) pg/ml], ***p < 0.001. There was no significant difference in the levels of NEP between groups AF-stroke.

The plasma level of Corin in the AF-stroke group [8.17 (4.68–30.62) ng/ml] was significantly higher than that in the no AF-stroke group [4.93 (2.12–9.01) ng/ml] (*p* < 0.05). The plasma levels of BNP were significantly higher in the AF-stroke group [269.85 (64.54–633.90) pg/ml] than in the no AF-stroke group [13.27 (0–72.12) pg/mL] (*p* < 0.001). No significant difference was observed in the plasma levels of NEP between the AF-stroke group [130.5 (8.63–274.88) pg/ml] and the no AF-stroke group [131.12 (3.57–253.94) pg/ml] (*p* > 0.05; Table 1 and Figure 2).

## Comparison of CpG Methylation Levels

The comparison of CpG methylation levels in the promoter region of the Corin protein gene between the AF-stroke and no AF-stroke groups is shown in Figure 1C. The average methylation level of each CpG site in each sample was estimated using the box plot + bee colony plot to show the methylation distribution of each region between the AF-stroke and no AF-stroke groups, and the difference between these two groups was analyzed by the Wilcox test. The levels of CpG methylation in the promoter region of the Corin protein gene were significantly lower in the AF-stroke group than that in the no AF-stroke group (*p* < 0.05).

## Discussion

Increasing age has been widely recognized as a major risk factor for ischemic stroke in patients with AF, which is about 1.4- to 3.3-fold (Liao et al., 2019). This phenomenon was confirmed in the baseline clinical characteristics of the participants. The average age of the AF-stroke group [73 (64.5–82.5) years] was significantly higher than that of the no AF-stroke group [65 (56.5–74) years]. AF-related stroke is associated with a higher mortality rate, more disability, longer hospitalization, and worse function recovery compared to the no-AF-related stroke (Liao et al., 2019), which is also confirmed in the baseline treatment in this study. The patients in the AF-stroke group were older, had a higher initial NIHSS score, and 90-day mRs than those in the no AF-stroke group.

Biological aging is the gradual, progressive decline in system integrity that causes morbidity and disability (Field et al., 2018; Belsky et al., 2022). Field et al. (2018) and Belsky et al. (2020, 2022) quantitated the pace of biological aging from a DNAm blood test. The vascular stiffness of the elderly population increases with aging. Vascular aging is a pivotal risk factor for dysfunction and related diseases. DNAm is involved in vascular aging and plays a central role in regulating age-related vascular diseases. Moreover, arterial stiffness and vascular aging trigger cerebrovascular dysfunction and constriction of the blood-brain barrier, leading to cerebrovascular diseases (Thorin-Trescases et al., 2018; Xu et al., 2021). The evaluation of vascular aging might aid in stroke risk assessment in a community-based Chinese cohort conducted with 11,474 participants (Yang et al., 2019). As described above, DNAm is a dynamically reversible process involving altered gene transcription without modifying the DNA sequence. It changes with age (Acton et al., 2021) and plays a critical role in vascular aging. Age-related CpG sites from blood samples based on DNAm can be used to construct the modeling tools to predict biological aging (Li et al., 2018).

Several studies demonstrated that DNAm regulates several cardiovascular and cerebrovascular diseases, such as cardiac remodeling, heart failure, atherosclerosis, stroke, dementia, and AD (Chen et al., 2021; Xu et al., 2021). Recent studies have highlighted the critical role of mechanosensory-related epigenetics in local endothelial dysfunction and regional susceptibility to vascular disease. DNA de/methylation promotes endothelial dysfunction in major arterial and venous diseases, leading to altered hemodynamics, which can be used as a biomarker in the early stages of vascular disease (Karthika et al., 2021). Based on these theories, we investigated whether DNAm enhances the understanding of vascular aging and related diseases. The current results showed that the characteristics of the AF-stroke group included older age, high severity, and poor prognosis, indicating that AF-related stroke may be associated with aging, while the no AF-stroke group was associated with factors associated with vascular aging, especially atherosclerosis risk factors (for example, hypertension, hypercholesterolemia, and smoking; Lakatta, 2008; Gomez-Marcos et al., 2016; Table 1 and Figure 2). This phenomenon may be attributed to the fact that the no AF-stroke group selected in our study exhibited the LAA-type stroke. The study showed that risk factors associated with vascular aging are associated with no AF-stroke and, therefore, may play a role in the pathogenesis of stroke in the no AF-stroke group. Next, we selected the four methylation sites with maximal differences between the groups: CORIN:678, CORIN:682, CORIN:694, and CORIN:700 (Figures 1A–C). The hypomethylation levels at these CpG sites serve as peripheral blood biomarkers for predicting AF-related stroke.

The concentration levels of each component of the NP family are critical to ensure proper control of systemic and local cardiovascular function. In order to achieve the ultimate optimal levels of NPs, a fine balance is required between gene expression, protein secretion, and clearance, with a key role of gene expression regulation and translation (Rubattu et al., 2020). The heterogeneous group of molecular biomarkers of AF encompasses the products of the neurohormonal cascade, including Corin–BNP–NEP. These biomarkers could be used for AF diagnosis and prediction of the transition from paroxysmal to persistent AF (Tsioufis et al., 2019). Hijazi et al. (2016) developed a novel biomarker-based risk score for predicting stroke in AF, termed the age, biomarkers, and clinical history (ABC) stroke risk score based on the independent association between NT-proBNP and the occurrence of AF-associated stroke. This finding suggested a strong association between the NP system and stroke associated with AF. Our prospective study showed that the plasma levels of Corin and BNP in the AF-stroke group were significantly higher than those in the no AF-stroke group (Table 1 and Figure 2). A previous meta-analysis suggested that elevated blood levels of natriuretic peptides (BNP/NT-proBNP) are repeatedly associated with cardioembolic stroke (Llombart et al., 2015). Fukuhara et al. (2020). demonstrated that BNP is inversely correlated with a favorable outcome of stroke if the estimation is within 24 h of stroke onset. These findings were also verified in the current study.

Corin is one of the major enzymes in the splicing of proBNP into BNP. Peng et al. (2015) found that the serum-soluble Corin level was lower in patients with stroke than in healthy controls, which further deemed a pathogenic role of serum-soluble Corin in stroke. Strikingly, the study did not measure NP levels, which could affect the activity of Corin. The follow-up study showed that serum-soluble Corin deficiency predicted major disability or death within 3 months after stroke onset, suggesting a probable role of serum-soluble Corin deficiency in stroke prognosis (Hu et al., 2016). A study by Chen demonstrated that plasma concentrations of Corin and NT-proBNP were significantly higher in patients with AF than in healthy controls (Chen et al., 2015), which could explain why the plasma concentrations of Corin and BNP were higher in the AF-stroke group than in the no AF-stroke group in our study.

Neprilysin is a ubiquitous membrane protease that is inactivated via the degradation of 40 peptides, including BNP (Bayes-Genis et al., 2016; Campbell, 2017). The current results did not show any significant difference in serum NEP levels between the AF-stroke and no AF-stroke groups (Table 1 and Figure 2), which has not been explained previously. It has been hypothesized that only the active form of NEP affects the BNP levels (Feygina et al., 2019). In a clinical trial, sacubitril/valsartan was used to intervene in the NEP activity of patients with heart failure. Consequently, increased concentrations of NEP substrates, such as atrial natriuretic peptide (ANP), substance P, and glucagon-like peptide 1 were observed, but no changes were detected in plasma BNP. On the other hand, the concentration of NT-proBNP decreased slightly (Nougué et al., 2019). This correlation between NEP and BNP might explain the above findings.

Nevertheless, the present study has some limitations. This was a single-center retrospective study, which might have selection bias. Due to the small sample size, the subsequent analysis may be limited. Since the current study population was of Asian descent, the results may not be applicable to other ethnic groups. Herein, we could not distinguish between paroxysmal, persistent, long-standing persistent, and permanent AF as Holter monitoring was not performed in this study. Also, the secondary outcomes, including mortality and hemorrhage, needed in future large-scale studies were not evaluated.

## Conclusion

In summary, the plasma-soluble Corin and BNP level was significantly higher in patients with the AF-stroke group than in the no AF-stroke group, while NEP was negative. The current findings raised the possibility that the Corin–BNP–NEP protein pathway is involved in the pathogenesis of AF-stroke. In addition, we also found that deficient CpG methylation in the promoter region of the Corin protein gene is associated with AF-related ischemic stroke.

## Data Availability Statement

The original contributions presented in the study are included in the article/supplementary material, further inquiries can be directed to the corresponding author/s.

## Ethics Statement

The studies involving human participants were reviewed and approved by the Ethics Committee of the First Hospital Affiliated to Soochow University. The patients/participants provided their written informed consent to participate in this study.

## Author Contributions

QF and XS conceived and designed the research. XS, ND, and YX analyzed the data and drafted the manuscript. LH, RY, JL, XZ, TX, YW, CC, ML, YJ, and LY collected the data and performed the research. All authors reviewed, edited the manuscript, and approved the final version of the manuscript.

## Conflict of Interest

The authors declare that the research was conducted in the absence of any commercial or financial relationships that could be construed as a potential conflict of interest.

## Publisher’s Note

All claims expressed in this article are solely those of the authors and do not necessarily represent those of their affiliated organizations, or those of the publisher, the editors and the reviewers. Any product that may be evaluated in this article, or claim that may be made by its manufacturer, is not guaranteed or endorsed by the publisher.

## Footnotes

1
http://www.R-project.org
